# Subcutaneous Injection of Oxyfluorfen Herbicide to the Forearm: Case Report

**DOI:** 10.1055/s-0037-1609048

**Published:** 2017-12-21

**Authors:** José Couceiro, Gonzalo Garcia-Portal, Olga Garcia

**Affiliations:** 1 Orthopedics Department, Hand Surgery Unit, Hospital Universitario Marques de Valdecilla, Santander, Cantabria, Spain; 2 Hand Surgery Unit, Department of Orthopedics, Hospital Univeristario Marques de Valdecilla, Santander, Cantabria, Spain; 3 Department of Pathology, Hospital Universitario Marques de Valdecilla, Santander, Cantabria, Spain

**Keywords:** oxyfluorfen, herbicide, injection, fasciectomy

## Abstract

**Background** Oxyfluorfen, a commercially available pesticide, commonly used for weed control in crop production, has been studied in terms of its toxicity, its carcinogenic properties, and its teratogenicity. We have found no reports, however, of the effects produced by an oxyfluorfen injection to the upper limb.

**Methods** We present the case of a 40 years old psychiatric patient, who reportedly injected her forearm accidentally while fumigating her garden. She was treated with irrigation and open forearm fasciectomy.

**Results** At 6 months, the patient had some tenderness at the scar; she wanted no further procedures done.

**Conclusion** Oxyfluorfen appeared to produce a chemical burn to the forearm tissues including the fascia, removal of the chemical product, and a limited fasciectomy, resulted in a favorable outcome.

Oxyfluorfen, a commercially available pesticide, commonly used for weed control in crop production, has been studied in terms of its toxicity, its carcinogenic properties, and its teratogenicity. We have found no reports, however, of the effects produced by an oxyfluorfen injection to the upper limb.

## Case Report

Our case is a 40 years old female patient, with a history of depression, following a process of complicated grief and dysthymia, associated with a period of alcohol consumption; she had been admitted to our psychiatry ward on four previous occasions, after different autolytic attempts.

**Fig. 1:**
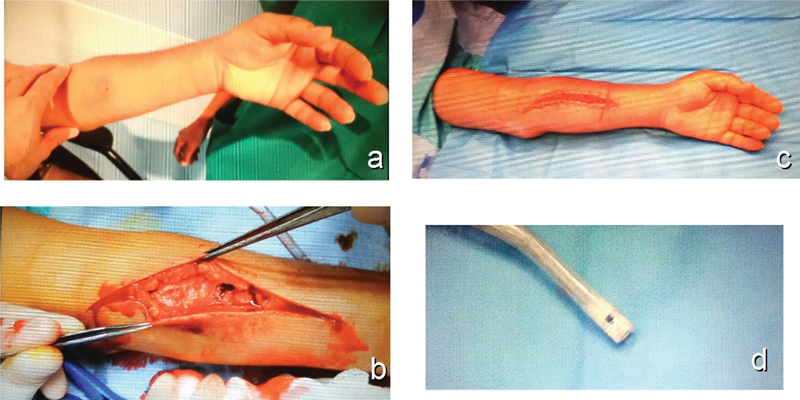
( **A** ) The puncture wound can be seen at the proximal forearm. ( **B** ) The fascia appeared white with a chalk-like consistence. ( **C** ) The wound was closed using a vessel—loop on a bootlace pattern. ( **D** ) The aspect of the author's vacuum system following surgery.

She consulted to our emergency department, declaring that she had sustained an injection of an undetermined quantity of herbicide (Oxyfluorfen, Goal, Dow AgroSciences, IN) to her left forearm. On her initial physical exam, her left forearm was swollen, and extremely painful on palpation. Her capillary refill was intact, and her two tips discrimination was 6 mm on all of her fingertips. There was a visible puncture wound on the proximal forearm ( Fig. 1A ). She reported no vomiting, her breathing was normal, and she did not have any other poisoning signs. Her ecographic exam showed a liquid layer overlying the fascial plane, but was otherwise unremarkable.

**Fig. 2:**
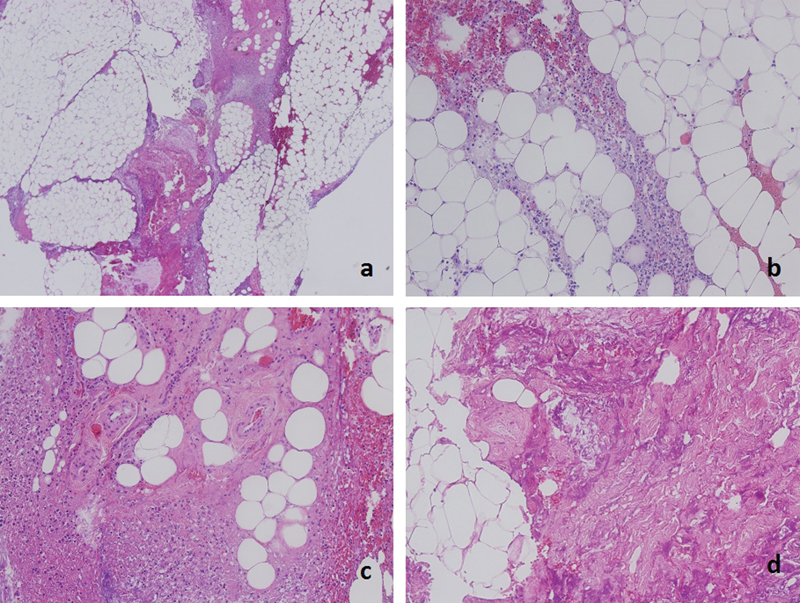
( **A** ) Hematoxilin and eosin stain 10x showing fat necrosis. ( **B** ) 20x Lipid vacuoles surrounded by inflammatory tissue. ( **C** ) 20x Normal appearing vessels with no signs of associated vasculitis. ( **D** ) 20x Extensive tissue necrosis.

The patient was taken for emergency surgery; a J-shaped approach was performed starting at the injection site, and progressing distally, the fascia and also the subcutaneous tissue appeared white, and had a chalk-like consistency ( Fig. 1B ). The remaining herbicide was drained from the forearm, and the affected fascia was excised. The incision was approximated using a vessel-loop following a bootlace pattern. Tissue samples were sent for analysis to the pathology department ( Fig. 2A–D ). The samples exhibited an extensive panniculitis and fat necrosis.

At 6 months follow-up, the incision was correctly healed, the patient́s neurological exam was unremarkable, and she had a correct capillary refill; she referred some tenderness at the surgical site. Her quick dash score was 13, her grip strength measured with a Jamar dynamometer (Bolingbrook, IL, USA) was 14 kg on the affected side and 20 kg on the unaffected side. She wanted no further procedures done.

## Discussion

Oxyfluorfen is a pre-emergent and postemergent liquid herbicide; it is a contact herbicide and light is required for it to affect the targeted plants. Oxyfluorfen's color ranges from yellow to orange, and it has an estimated pH of 7.11. It has been described as moderately toxic by ingestion and slightly toxic by dermal absorption. It is advised not to breathe the vapors or the nebulization, and to prevent the contact of the substance with the skin, the clothing, and the eyes. The manufacturer includes special recommendations for firefighting; when oxyfluorfen burns, the smoke may contain toxic unidentified components, nitrogen oxide, hydrogen fluoride, or hydrogen chloride among others; the use of a self-contained breathing apparatus is recommended under these circumstances. 1 There appears to be so far no data available on toxicity following a subcutaneous injection of this substance.

We have found no previous reports on oxyfluorfen injections to the forearm or hand. There are isolated case reports of other herbicide injections such as paraquat 2 3 4 and glyphosate-surfactant. 5 Paraquat injections appear to be one of the best documented ones; it is considered to be fatal if inhaled and harmful if swallowed. 6 On a classic article, Almog and Tal 2 reported a case of paraquat subcutaneous injection, the patient, a farmer with a history of paranoid schizophrenia, had injected himself with 1 mL of a 20% paraquat solution. He was admitted to the hospital after vomiting and passing bloody stools. The patient died 18 days after admission. In 2012, Gheshlaghi et al 4 published a case report, describing a deliberate intramuscular injection of 15 mL of paraquat solution. The patient a 25 years old lady died 41 hours after injection. It is of note that none of the two cases underwent any surgical debridement procedures. Fernando 3 reports a case of survival following a subcutaneous paraquat injection; the patient was a 19 years old girl who had quarreled with her parents. She underwent immediate surgical debridement; the affected skin and subcutaneous tissue were excised; the patient's clinical course was uneventful, and she was discharged from the hospital after 7 days.

In our case, we found that oxyfluorfen produced a chemical burn to the fascia and subcutaneous tissue of the forearm; we performed an emergent partial fasciectomy to eliminate as much of the diseased tissue as possible. We agree that the best course of action for these cases appears to involve an early extensive surgical debridement as suggested by Fernando. 3 Although it is likely that the vaporized herbicide dose is far below the toxic threshold, the authors think, judging by the state of their vacuum system after surgery, that it is advisable to take extreme precautions, such as to use ocular protection and face masks with particle filters.
